# The use of Bayesian methodology in the development and validation of a tiered assessment approach towards prediction of rat acute oral toxicity

**DOI:** 10.1007/s00204-021-03205-x

**Published:** 2022-01-16

**Authors:** James W. Firman, Mark T. D. Cronin, Philip H. Rowe, Elizaveta Semenova, John E. Doe

**Affiliations:** 1 School of Pharmacy and Biomolecular Sciences, Liverpool John Moores University, Liverpool, UK; 2 Department of Mathematics, Imperial College London, London, UK

**Keywords:** New approach methodologies, Bayesian inference, Tiered assessment, Regulatory toxicology, Acute toxicity, In silico toxicology

## Abstract

There exists consensus that the traditional means by which safety of chemicals is assessed—namely through reliance upon apical outcomes obtained following in vivo testing—is increasingly unfit for purpose. Whilst efforts in development of suitable alternatives continue, few have achieved levels of robustness required for regulatory acceptance. An array of “new approach methodologies” (NAM) for determining toxic effect, spanning in vitro and in silico spheres, have by now emerged. It has been suggested, intuitively, that combining data obtained from across these sources might serve to enhance overall confidence in derived judgment. This concept may be formalised in the “tiered assessment” approach, whereby evidence gathered through a sequential NAM testing strategy is exploited so to infer the properties of a compound of interest. Our intention has been to provide an illustration of how such a scheme might be developed and applied within a practical setting—adopting for this purpose the endpoint of rat acute oral lethality. Bayesian statistical inference is drawn upon to enable quantification of degree of confidence that a substance might ultimately belong to one of five LD50-associated toxicity categories. Informing this is evidence acquired both from existing in silico and in vitro resources, alongside a purposely-constructed random forest model and structural alert set. Results indicate that the combination of in silico methodologies provides moderately conservative estimations of hazard, conducive for application in safety assessment, and for which levels of certainty are defined. Accordingly, scope for potential extension of approach to further toxicological endpoints is demonstrated.

**Supplementary Information:**

The online version contains supplementary material available at 10.1007/s00204-021-03205-x.

## Introduction

The publication of “Toxicity Testing in the 21st Century” set an expectation that the manner in which chemical safety is assessed with regards to human health would soon radically change (Krewski et al. 2010). Since that time, many initiatives have emerged based upon adoption of novel methodologies (broadly termed New Approach Methodologies, or NAM) (Dal Negro et al. 2018). However, the bulk of regulatory decision-making, a fifth of the way through the twenty-first century, remains grounded in traditional laboratory animal-based techniques developed in the third quarter of the 20th. Regulators in the 1970s would not have been content with making use of methods devised in the 1920s, so why should we in the 2020s continue to rely on strategies similarly developed half-a-century ago?

Many NAM have been developed, both in vitro and in silico, with the aim of assessing biological effect, thereby enabling extrapolation to the in vivo sphere. The former includes a plethora of biomaterial-based systems incorporating either whole cells or their components (Anadón et al. 2014), whereas the latter encompasses techniques spanning quantitative structure–activity relationship (QSAR) modelling, machine learning and the creation of structural alerts (Madden et al. 2020). The rationalisation of NAM findings may be underpinned by concepts such as the Adverse Outcome Pathway (AOP)—which charts mode and mechanism of toxic action through stages including molecular initiating and key events (typically cellular), to ultimate adverse outcome at the organ and organism-level (Edwards et al. 2016; Tollefsen et al. 2014; Vinken 2013). Despite such progress, regulatory adoption of new approaches has remained slow (Knight et al. 2021). Whilst many validation programmes have been attempted, a lack of confidence in the general applicability of NAM continues to exist (Parish et al. 2020). We postulate that there are at least two fundamental reasons behind the persistence of this sense of scepticism: potential flaws within the methodologies themselves, and concerns regarding the lack of a means towards validation. Both factors may contribute towards lower acceptability for regulatory use (Mahony et al. 2020).

The twin factors of flawed methodology and inappropriate validation are interlinked. Initially, in the search for techniques not involving the use of laboratory animals, individual approaches were assessed directly for their ability to reproduce the results of the in vivo protocol which they were intended to replace (Piersma et al. 2018). Outcomes of single tests were compared against those of animal studies, to gauge the number “correct” answers matched. This was often performed using a sensitivity/specificity paradigm based upon a simplistic conception of “wrong and right answers”, in turn leading to concerns over the occurrence of false positive and false negative results. There are issues with this approach. Firstly, given the level of complexity entailed, it is unrealistic to expect that a single test might reliably reproduce the results of an animal study: a combination of sources shall more likely produce success (Piersma et al. 2014). Secondly, there is very little in toxicology which is truly binary: a continuum is usually present, with “positive” and “negative” judgments dictated by the side of a line on which the answer lies.

The concept of compounding data derived from an assortment of in silico, in vitro and in vivo sources, to produce a tiered assessment of toxic potential, has in recent years been advanced as a means of addressing each of these broad issues (Andersen et al. 2019; Thomas et al. 2013, 2019). Such an approach may not merely allow for the enhancement of confidence in prediction relative to isolated NAM (through intensifying weight-of-evidence), but may further be adapted to facilitate quantitative expression of certainty—extending resolution beyond the simple binary call. For this to be realised, a statistical methodology must be adopted through which the outputs of the various approaches can be integrated to produce updated judgments. Bayesian inference represents a powerful technique for achieving this, permitting as it does the generation of probabilistic distributions which may be related to severity of toxicity (Lazic and Williams 2021). Its application within predictive toxicology constitutes an emerging field of interest, and as such it has been drawn upon in recent studies aimed towards development of models describing endpoints spanning skin sensitivity (Reynolds et al. 2019), drug-induced liver injury (Semenova et al. 2020; Williams et al. 2020) and cardiotoxicity (Felli and Leishman 2020).

This paper describes a route towards the development and application of such a tiered approach – adopting, for illustrative purposes, the assessment of acute oral lethality within the rat. Bayesian methodology is employed to compute distributions relating the probability that a given substance might belong to one of five categories, each corresponding to a defined LD50 range mirroring those adopted within European Union Classification, Labelling and Packaging (CLP) regulation. Three tiers are incorporated, over which a variety of in silico and in vitro methodologies are exploited. In Bayesian terms, the outputs from the previous tier are adopted as the “prior” to inform that which follows. Analysis of predictive quality following introduction of each tier enables the certainty of its outcomes to be determined, in turn allowing for the contribution of the constituent techniques to be discerned. Alongside the sourcing of data from existing QSAR schemes, the training of novel machine learning algorithms and structural alert sets are reported. In addition to validating the tiered approach developed, our intention is to demonstrate how the output from such a system may be appropriately evaluated. Through this, it should be possible to ascertain whether or not the strategy is ultimately successful in its aims of improving, in principle, the acceptability of predictions derived from NAM.

## Materials and methods

### Sourcing of rat acute oral toxicity data

From the publication of Gadaleta et al. (2019), data describing acute oral toxicity towards rats within an inventory of organic substances were acquired. These data are themselves drawn from a yet wider selection collated through efforts of NICEATM and US EPA (https://ntp.niehs.nih.gov/go/tox-models; accessed 1–5–2020)—with final LD50 quantities relating to compounds possessing greater than three distinct point estimates being derived in accordance with methodology outlined by Nelms et al. (2020). Contained within the Gadaleta et al. dataset were records describing 11,363 substances, of which 8448 contained accompanying values relating experimental toxicity (LD50, expressed as mg/kg_bw_). Through removal both of duplicate entries and of those possessing undefined chemical structure (including mixtures and polymers), a final working set consisting of 8186 distinct organic molecules was formulated. Existing SMILES (www.daylight.com) (Weininger 1988) strings were retained, whilst chemical names were related to supplied CASRN either through use of the US EPA CompTox Chemicals Dashboard (https://comptox.epa.gov/dashboard; accessed 1–5–2020) (Williams et al. 2017) or through PubChem (https://pubchem.ncbi.nlm.nih.gov/; accessed 1–5–2020). For purposes of model development, LD50 estimates were transformed directly into their log mmol/kg_bw_ equivalents. Data are presented in full within Supplementary Table 1.

### Assignment of acute toxicity category to compounds

In accordance with the scheme outlined within Table 1, chemicals were each assigned a numerical acute oral toxicity category (1–5) derived directly from acute oral LD50. This rubric is itself adapted from that specified within European Council Regulation No. 1272/2008 (European Union 2008)—a minor amendment being the appending of a “Category 5”, covering compounds holding LD50 greater than 2000 mg/kg_bw_.

**Table 1:** Overview of scheme through which acute toxicity category is assigned from oral LD50

Acute tox. category	LD50 range (mg/kg_bw_)
1	< 5
2	5–49
3	50–299
4	300–1999
5	≥ 2000

### Derivation of Cramer scheme classification

The Cramer classification scheme assigns molecules into one of three “threshold of toxicological concern (TTC)” classes, describing broadly and conservatively their level of hazard towards human health under a repeat-exposure scenario (EFSA Scientific Committee 2019; Cramer et al. 1978). It is constituted as a decision tree, with presence or absence of characteristic chemical structural features determining the final placement of a compound. Class I corresponds to substances of least apparent toxic concern, Class II to substances of intermediate concern and Class III to substances of greatest concern. Cramer classifications for each compound within the working set were sourced though use of the “Cramer rules, with extensions” decision tree (Patlewicz et al. 2008), as implemented within ToxTree software (v. 3.1.0; EU Joint Research Centre and IDEAconsult; http://toxtree.sourceforge.net/). Classes were definitively assigned to 8180 of the 8186 chemicals within.

It was necessary that probability distributions were derived describing the relationship associating assigned classification with experimentally derived acute toxicity category. Raw distributions were initially acquired, based upon a simple count of the occurrence of toxicity categories amongst those compounds sharing a class. By way of illustration, amongst the 7097 substances assigned Class III, 219 (3.1%) fell into Category 1 based upon their in vivo LD50, 629 (8.9%) Category 2, 1385 (19.5%) Category 3, 3003 (42.3%) Category 4 and 1861 (26.2%) Category 5. However, adjustments were required on account of the marked imbalance present within the spread of experimental toxicity across the 8136 compounds forming the dataset. To achieve this, these raw counts were scaled in proportion to the occurrence of each category within experimental data (listed within Table 4, column “Distribution”). Given that 40.6% of compounds fell into Category 4 and 2.7% into Category 1, raw percentages for each were divided respectively by 0.406 and 0.027. Applying this to all categories, final adjusted distributions were acquired.

### Prediction of acute rat oral LD50 through use of US EPA Toxicity Estimation Tool

Contained within the US EPA Toxicity Estimation Tool (TEST) (v. 4.2.1; US EPA; https://www.epa.gov/chemical-research/toxicity-estimation-software-tool-test) is a variety of QSAR models relating directly to assignment of acute oral LD50 within the rat. Through use of the “hierarchical clustering” method, LD50 predictions could be acquired for 7479 of the 8186 screened substances. A detailed description of the methodology underlying this QSAR may be found within Martin et al. (2008).

### Development of random forest model for estimation of acute rat oral LD50

Relationship between chemical structure, calculated molecular physicochemical properties and corresponding acute oral LD50 was further modelled through the training of a random forest algorithm. Employed as input variables were a combination of molecular fingerprint fragments and physicochemical descriptors. Screening of compounds for their PubChem substructure fingerprint (ftp://ftp.ncbi.nlm.nih.gov/pubchem/specifications/pubchem_fingerprints.txt) was performed within KNIME software (v. 4.3.1; www.knime.com) (Berthold et al. 2008), through use of the RDKit (v. 2020.03.6; www.rdkit.org) (Landrum 2006) Fingerprint node. Fragments present in fewer than 5% or in greater than 95% of compounds were excluded from consideration, whilst all others (totalling 300) were retained. An assortment of 1D and 2D Molecular physicochemical descriptors were sourced from Molecular Operating Environment (MOE) software (v. 2018.01; Chemical Computing Group; https://www.chemcomp.com/) (Molecular Operating Environment 2018). Following removal of parameters unsuitable for modelling, a sum of 184 descriptors remained. Pooling with the aforementioned fingerprint fragments produced a set of 484 variables, characterising the complete collection of 8186 compounds. This full parameter set is available from the authors upon request. Employing log mmol/kg_bw_ LD50 as the modelled quantity, a random forest consisting of 500 trees was constructed within R (v. 4.0.1; R Foundation; https://www.r-project.org/) (R Core Team 2021), through use of RStudio (https://www.rstudio.com/) (RStudio Team 2020) and the randomForest package (Liaw and Wiener 2002). Applying ten-fold cross-validation, predictions for all modelled compounds were obtained.

### Compilation of molecular structural alerts associated with elevated acute oral toxicity

A series of tailored chemical structural alerts were developed, to assess the broader utility of the approach in enhancing the correct identification of potentially hazardous compounds—notably, those for which corresponding toxicity in vivo is liable to be understated through alternative methods such as QSAR or in vitro cytotoxicity screening. Such groups were identified through manual analysis of output from TEST hierarchical clustering, random forest and in vitro systems—and further rationalised through application of expert judgment. Broadly, the highlighted structures could be considered to fall into one of two varieties: those integral within mediating a mechanism of toxic action not reliably recapitulated within simple 2D cell-based assay systems (such as neurotoxicity or anticoagulant effect), or those representative of specific compound classes (typically complex natural products) not adequately modelled through global QSAR.

For those alerts which were present within a minimum of ten compounds, distributions relating the probability of a substance falling within a given acute toxicity category were determined through a method similar to that described within 2.3 (with raw counts scaled in proportion to the prevalence of each category within the experimental data). In several instances, the fragment forming the alert occurs within fewer than ten compounds across the dataset. Distributions in such cases are manually estimated, with probability of falling within Category 1 assigned at 90%, Category 4 at 10% and the remaining categories each 0%.

### Acquisition of in vitro cytotoxicity data

Data relating to cytotoxicity in vitro were gathered initially from a variety of sources, including the publications of Clothier et al. (2008), Prieto et al. (2013) and Kinsner-Ovaskainen et al. (2013)*.* However, owing to the very limited overlap apparent between the compounds present within these studies and those comprising the LD50 dataset (data not shown), their use was deemed impractical.

Cell viability assays present across the ToxCast (Richard et al. 2016) and Tox21 (Tice et al. 2013) platforms (accessed through US EPA CompTox Chemicals Dashboard) were subsequently examined for their suitability, in terms both of raw coverage and quality of correlation of AC50 with acute in vivo LD50. Assay “TOX21_RT_HEK293_GLO_16HR_VIABILITY” was found to be optimal in this regard (data not shown), covering a total of 543 substances. In brief, the technique assessed viability of HEK-293 (human embryonic kidney) cells following 16 h treatment with substance of interest, though a bioluminometric protocol.

### Overview of tiered approach

The aforementioned methodologies were portioned into one of three “assessment tiers”, each representing distinct approaches towards hazard prediction (Cramer classification, in silico and in vitro—as outlined within Table 2). Further reference is made within to a hypothetical “Tier 3”, in which it is envisioned that a refined in vivo protocol, such as that exemplified by OECD Test Guideline 425 (OECD 2008), would be selectively initiated to address uncertainties existing in category assignment towards specific compounds (an idea largely beyond the scope of this study, yet considered further within Discussion). The concordance of predicted and experimentally determined acute toxicity categories were assessed following application of each tier, to validate the contribution of each towards improving (or otherwise) the quality of forecast—both in terms of the proportion of compounds correctly assigned, and of those either under- or overpredicted. Details of the Bayesian model employed in attributing categories within Tier 1 and Tier 2 are provided within section “Bayesian analysis”.

**Table 2:** Overview of assessment tiers, outlining the composition of each in relation to methodologies incorporated

Assessment tier	Approach overview	Methodologies incorporated	Category assignment
0	Cramer classification	Cramer scheme (with extensions)	Classification-category probability distribution (Table 5)
1	In silico	EPA TEST LD50	Bayesian model
		Random forest LD50	
		Structural alerts	
2	In vitro	In vitro cytotoxicity (cell viability assay)	Bayesian model
3 (hypothetical)	In vivo	OECD Test Guideline 425: Acute oral toxicity	Experimental outcome

### Application of tiered approach

The tiered predictive approach was subsequently applied to a representative selection of 50 compounds from out of the 8186 forming the working dataset. On account of the inherent imbalance present within the distribution of toxicity across the unfiltered set, it was necessary to ensure that these representatives were drawn in equivalent numbers from each of the five acute toxicity categories. To meet criteria for inclusion, substances had to have received a valid Cramer classification, LD50 assignments through both EPA TEST and random forest techniques, and data from the “TOX21_RT_HEK293_GLO_16HR_VIABILITY” assay. Presence of a structural alert was, however, not a requirement.

A total of 496 molecules were found to meet the above stipulations. Ten each were drawn from Categories 3, 4 and 5. Since only six eligible substances were observed to fall into Category 1, an additional four were drawn from amongst those in Category 2 to account for shortfall. The composition of this selection may be found within Supplementary Table 2.

### Bayesian analysis

Bayesian predictive models were constructed based upon proportional odds logistic regression (POLR). The outcome variable *y*_*i*_ is represented by ordered categorical data, i.e. compound’s acute toxicity category. For each compound *i* the model calculates the underlying continuous severity *η*_*i*_ based on available predictors *X*_*i*_ using a set of regression coefficients *β*_*i*_. A set of cut-points *c*_1_,..,* c*_4_ subdivides the continuous severity to define boundaries between five discrete ordered categories. Regression coefficients and cut-points are inferred from data. Tier 1 model incorporates the distribution emerging from Tier 0, updating with outputs acquired from EPA TEST, random forest and (as applicable) from matched structural alerts. Subsequently, this is brought forward to Tier 2—whereby in vitro data is integrated to provide the ultimate posterior.

The Bayesian approach to model fitting requires specification of a likelihood and priors for each parameter. We adopt the priors from Williams et al. (2020), i.e. the regression coefficients are described by a Laplace distribution with mean *μ* and standard deviation *σ*; *μ* and *σ* are the hyperparameters of the model and are also estimated by the model. The cut-points are assigned weakly informative normal priors with mean 0 and standard deviation 20. We used RStan interface (v. 2.21.2; http://mc-stan.org/) (Stan Development Team 2020) of the Stan modelling language (Carpenter et al. 2017). Predictions were derived from the samples obtained from four chains, 10,000 iterations each of the No-U-Turn sampling algorithm.

### Category attribution (exclusionary method)

Assignment of predicted acute toxicity category was performed based upon categorical probability distributions obtained following application of each respective tier. In the instance of Tier 0, this was the scaled probability distributions derived from Cramer classification. Bayesian models drawn from data presented within Tiers 1 and 2 produced corresponding outputs which were treated identically. For a given compound, all categories for which probability of belonging fell beneath a specified threshold (be it 10% or 5%) were excluded from consideration (termed “exclusionary method”). Compounds were then attributed to the remaining category indicating the greatest hazard. This tended to produce conservative allocations, which would be preferable for purposes of risk assessment. By way of illustration, consider compounds holding distributions outlined within Table 3: applying a 10% threshold to Compound A leads to the exclusion of all with the exception of Categories 2 and 3. It is Category 2, representing as it does greatest toxicity, which is assigned. Optional lowering of the threshold to 5% expands the remit to incorporate Category 1—which is accordingly granted. Compounds B and C provide further example of the approach in action.

**Table 3:** Category-probability distributions relating to three hypothetical compounds—A, B and C

	Scaled probability distribution (%)					Category assignment	
	Acute toxicity category					Threshold (%)	
	1	2	3	4	5	5.0	10.0
Compound A	8.0	55.0	30.0	4.0	3.0	1	2
Compound B	0.0	5.0	15.0	50.0	30.0	2	3
Compound C	0.0	0.0	1.0	9.0	90.0	4	5

Acute toxicity categories, assigned following application of exclusionary thresholds 5.0% and 10.0%, are listed

## Results

### Overview of data landscape

Application of the acute toxicity categorisation scheme described within section “Assignment of acute toxicity category to compounds” to the collection of 8136 compounds possessing rat acute oral LD50 data produced the distribution outlined through Table 4. As is apparent, greater than 70% of substances possessed toxicity sufficient to place them within the two categories of least concern (4 and 5). By contrast, only approximately 10% fell within those representing the greatest hazard (1 and 2)—notable being neurotoxicants of the organophosphate and carbamate classes, vitamin K-antagonist anticoagulants and various natural products (such as strychnine and saxitoxin) representative of wider toxin families.

It is informative to note that, although the in vivo experimental LD50 values described above are presented as point estimates, they are in fact the medians of a probability distribution. LD50 is usually determined through use of a probit calculation derived from lethality dose–response, with the median taken as a final value. In a prior analysis of this dataset (Kleinstreuer et al. 2018), it was reported that 28% of member chemicals possessed greater than two values corresponding to point estimates of acute oral LD50. This enabled evaluation of the variation in LD50 which ideally must be considered when framing concordance between the experimental LD50 and results derived from the tiered approach. It was determined that the 95% confidence limit for LD50 values was ± 0.31 log_10_ (mg/kg). It is thus illuminating to compare the calculated 95% confidence limits for the LD50 values, which is equivalent to a probability range of 0.62 log_10_ (mg/kg), with the ranges for categories 2–4 which have both upper and lower limits. The limits for the classification categories are themselves 0.8 or 1 log_10_ (mg/kg), and so it is likely that some chemicals with a single value would fall into a different category were the study to be repeated. Confusion matrices (Supplementary Table 3) were constructed to assess the impact of adding or subtracting 0.31 log_10_ (mg/kg) to the experimental value for the balanced subset of 50 LD50 values used to evaluate the tiered approach. It can be seen that 32% of all chemicals changed category when the confidence limit was added, whereas 24% switched when the limit was instead subtracted.

**Table 4:** Distribution of compounds in accordance with acute toxicity category occupied—expressed in terms both of raw quantity and of percentage of total

Acute tox. category	LD50 range (mg/kg_bw_)	Number of compounds	Distribution (%)
1	< 5	219	2.7
2	5–49	638	7.8
3	50–299	1452	17.7
4	300–1999	3326	40.6
5	≥ 2000	2551	31.2

### Cramer classification

Scaled probability distributions related to Cramer classification are displayed within Table 5. As may be seen, the great majority of compounds were assigned Class III—that representing highest concern with respect to repeat-dose toxicity. Across those compounds falling within Classes I and II, clear trends are observed highlighting a similar, transferable association with reduced acute toxicity. Substances matching either call display steadily increasing probabilities of belonging to higher, more inert categories. This should be contrasted with Class III, for which the corresponding distribution is essentially flat.

**Table 5:** Scaled category-probability distributions derived from Cramer classification

	Scaled probability distribution (%)					Dataset coverage (%)
	Acute toxicity category					
	1	2	3	4	5	
Cramer classification						
I	0.0	2.6	10.2	22.3	64.8	11.4
II	0.0	7.6	13.3	25.2	53.8	1.9
III	21.9	21.6	20.9	19.7	16.0	86.7

### EPA test

**Fig. 1:**
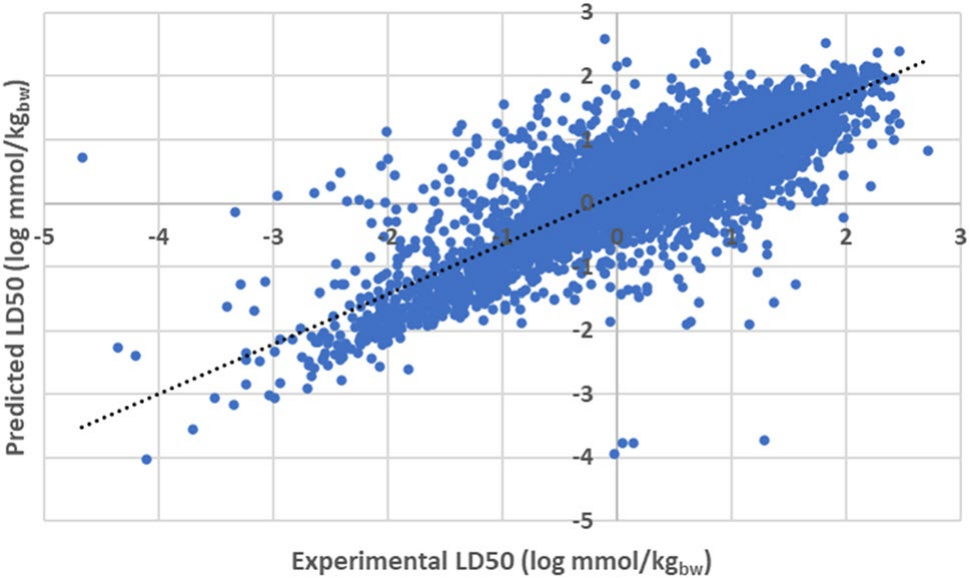
Plot outlining correlation between acute oral LD50 as predicted through EPA TEST hierarchical clustering model, and that determined experimentally (*r*^2^ = 0.739)

Predicted acute oral LD50 values (covering 7479 compounds) sourced using the US EPA TEST hierarchical clustering model were found to, in general, correlate well with experimental quantities (*r*^2^ of 0.739). Relationship is depicted graphically within Fig. 1.

### Random forest

**Fig. 2:**
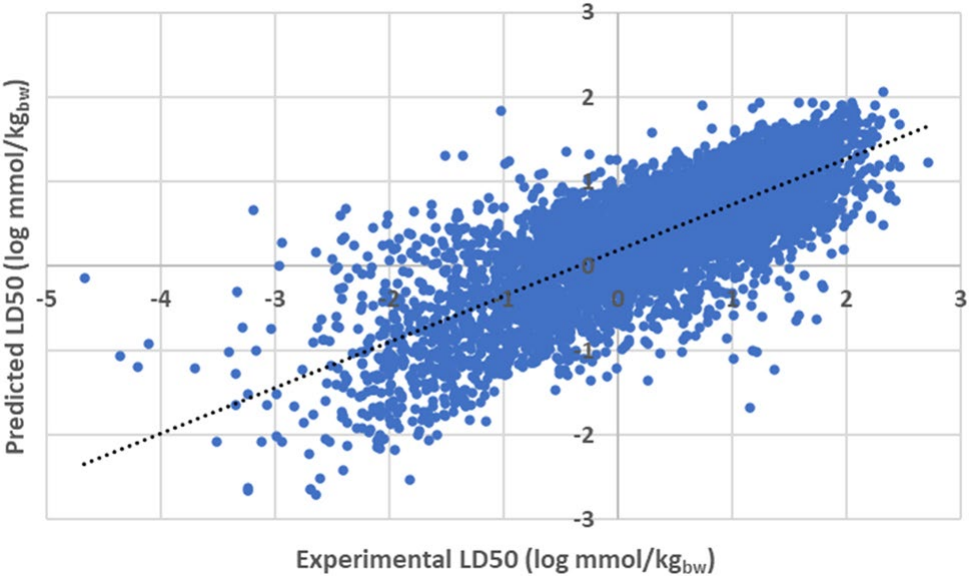
Plot outlining correlation between acute oral LD50 as predicted through random forest model, and that determined experimentally (*r*^2^ = 0.602)

The performance of the random forest model in the prediction of LD50 was, overall, inferior to that of EPA TEST (*r*^2^ of 0.602). Relationship is depicted graphically within Fig. 2.

### Structural alerts

A total of thirteen structural alerts were formulated, highlighting molecular fragments represented notably within compounds prevalent across acute toxicity categories 1 and 2. Dependent upon the absolute frequency of their occurrence within the dataset, category-probability distributions for each were either calculated or estimated. Details relating to those five features present with sufficient coverage so that their distributions were calculated may be found within Table 6.

**Table 6:** Overview of structural alerts present within ten or greater compounds

Alert title	Defining structure	Mechanistic considerations	Scaled prob. distribution (%)					Coverage
			Acute toxicity category					
			1	2	3	4	5	
Organophosphate		Neurotoxin (acetylcholinesterase inhibition)	41.8	33.9	16.7	4.6	3.1	730
Carbamate		Neurotoxin (acetylcholinesterase inhibition)	31.5	40.0	16.4	8.3	4.2	327
Fluoromethyl-benzimidazole (fenazaflor-like)		Apparent inhibition of oxidative phosphorylation	51.6	41.3	7.0	0.0	0.0	128
Vitamin K antagonist (warfarin-like)		Anti-coagulant	84.4	5.8	7.6	2.2	0.0	11
Dibenzodioxin		Uncertain	96.3	3.7	0.0	0.0	0.0	10

Depicted is the defining structural fragment, alongside details relating to the known toxic mechanism associated with the class, its scaled category-probability distribution and its absolute coverage

Eight further alerts were identified—representative of those classes associated with high oral toxicity, yet present only sparsely within the working dataset (fewer than ten compounds). Six of these described the core structural features of complex natural product families: aflatoxins, ochratoxins, saxitoxins, strychnine, trichothecenes and vitamins D. The remaining two corresponded, respectively to synthetic bromethalin neurotoxicants and indandione anticoagulants. Expanded description of all is presented within Supplementary Table 4.

### Cytotoxicity in vitro

**Fig. 3:**
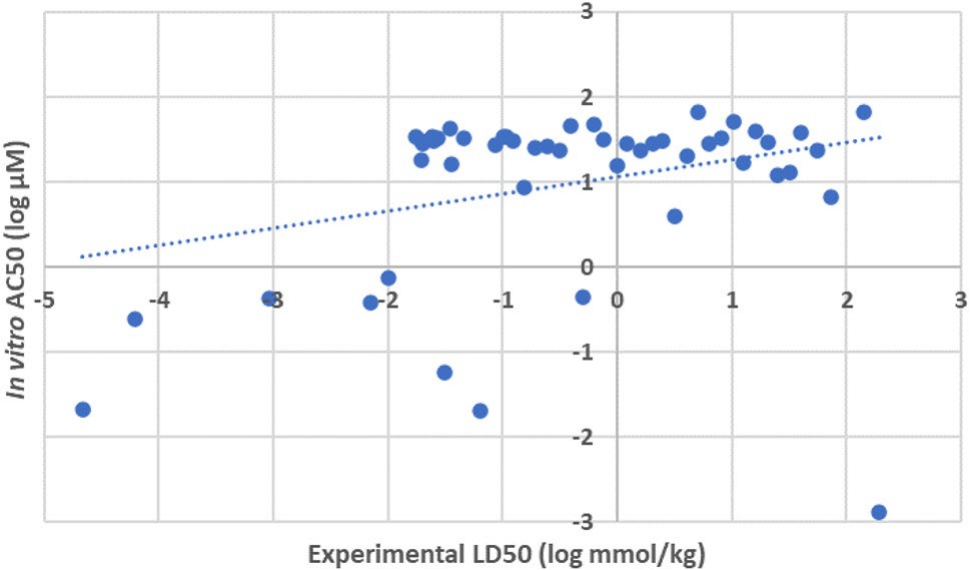
Plot outlining correlation between in vitro cytotoxicity (expressed as log AC50) and acute oral LD50 (*r*^2^ = 0.0877)

Of the variety of ToxCast cell viability assays examined, none were found to provide meaningful concordance with experimental acute oral LD50 (as noted within Section “Acquisition of in vitro cytotoxicity data”). Across the 50 compounds forming the balanced representative set, the settled-upon assay (TOX21_RT_HEK293_GLO_16HR_VIABILITY) produced a correlation with *r*^2^ merely 0.0877. Figure 3 depicts this relationship graphically.

### Tiered approach

Each of the 50 compounds selected to form a balanced, representative sample of the larger dataset (refer to Supplementary Table 2) were passed through the series of assessment tiers outlined within section “Overview of tiered approach”. Chosen primarily on account of the range of toxicity spanned, the sample cohort furthermore exhibited diversity with respect to chemical space and functional use—from comparatively simple molecules such as 4-tert-butyltoluene and 2-methylbutanal to complex natural products emetine and digitoxin. Examples of pharmaceuticals, endogenous biomolecules, synthetic intermediates and pesticides were each present.

#### Tier zero: Cramer classification

**Fig. 4:**
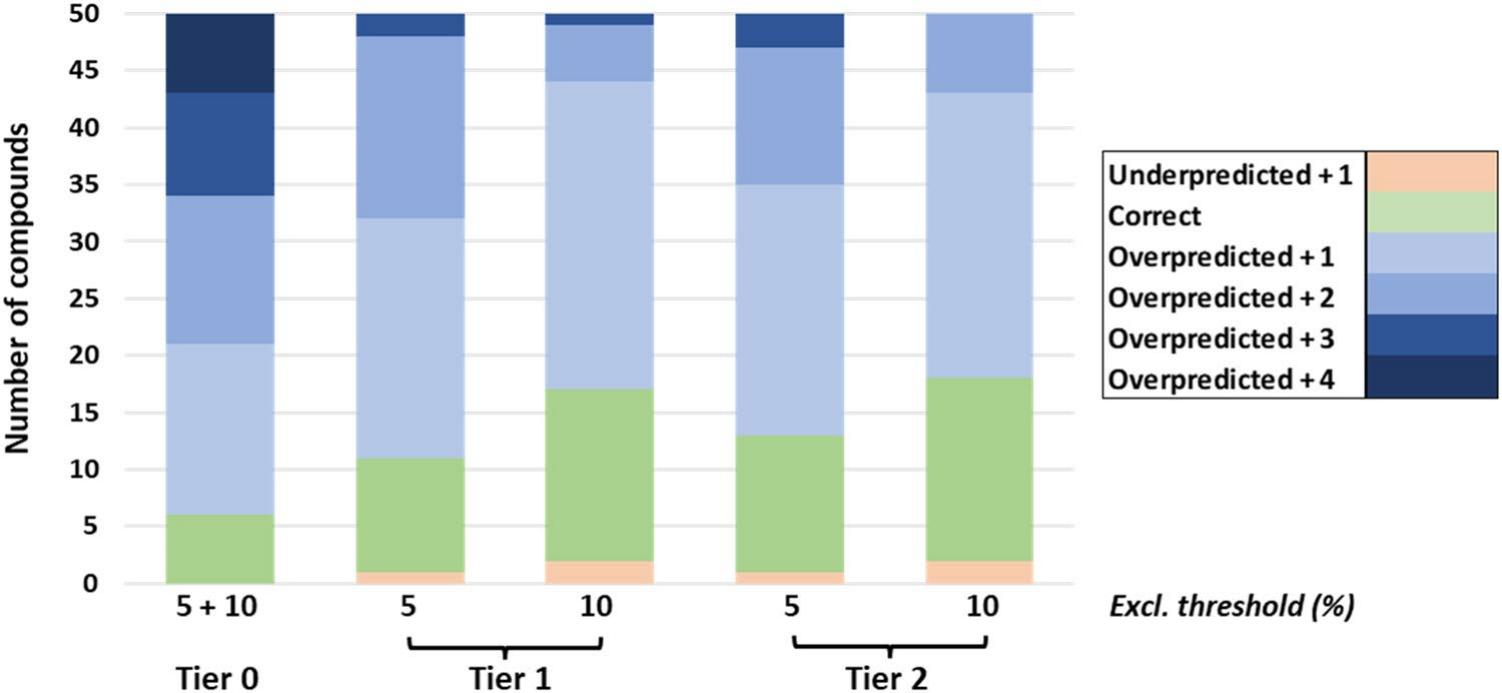
Variation in extent of deviation in category assignment relative to experimentally determined classification, in accordance with assessment tier and exclusionary threshold. Colouration relates to extent of over- or underprediction, defined as differential between predicted and experimental toxicity categories

Applying the exclusionary method (see section “Category attribution (exclusionary method)”) to the Cramer classification-derived probability distributions presented within Table 5 produced overly conservative estimations of acute toxicity category (presented in the form of a confusion matrix applicable to both 5% and 10% thresholds—Table 7). As is expressed within Fig. 4, 44 compounds from 50 were predicted to hold categories indicative of higher toxicity than that which they exhibit in vivo—that is, they are overpredicted. This is largely a consequence of the automatic assignment of Cramer class III compounds (46 from 50, including nine Category 4 and seven Category 5) to toxicity Category 1. Given the extremity of the toxicity represented by such a label, it is inevitable that this relationship shall, in the great majority of instances, produce marked overstatement of associated hazard. The four compounds falling into Cramer class I were each attributed Category 3—again, a cautious assessment of their experimentally-defined toxicity.

**Table 7:** Confusion matrix outlining category assignment following application of Tier 0 (applicable both to exclusionary thresholds 5.0% and 10.0%)

		Category (predicted)				
		1	2	3	4	5
Category (experimental)	1	6	0	0	0	0
	2	14	0	0	0	0
	3	10	0	0	0	0
	4	9	0	1	0	0
	5	7	0	3	0	0

#### Tier one: in silico (EPA TEST + random forest + structural alerts)

Through adoption of a Bayesian statistical approach (as outlined within section “Bayesian analysis”), Cramer classification-derived distributions were supplemented with outcomes from the aforementioned suite of in silico techniques: EPA TEST, random forest and structural alerts. Their effect, as anticipated, was to mitigate against the excessively conservative nature of the Tier 0 assignments—producing a series of category-level predictions exhibiting a far greater degree of resolution and balance. Extent of overprediction was noted to fall, whilst instance of correct assignment grew (see matrices presented within Table 8).

**Table 8:** Confusion matrices outlining category assignment following application of Tier 1, following application of exclusionary thresholds 5.0% (a) and 10.0% (b)

		Category (predicted)				
		1	2	3	4	5
(a) Category (experimental)	1	5	1	0	0	0
	2	10	4	0	0	0
	3	5	5	0	0	0
	4	0	7	2	1	0
	5	0	2	4	4	0

		Category (predicted)				
		1	2	3	4	5
(b) Category (experimental)	1	4	2	0	0	0
	2	8	6	0	0	0
	3	1	8	1	0	0
	4	0	1	7	2	0
	5	0	1	3	4	2

Comparing within-tier, the inherent enhanced conservatism of the 5.0% exclusionary threshold relative to 10.0% was readily apparent. With application of the 5.0% threshold, quantity of compounds for which toxicity category was predicted correctly rose from six (in Tier 0) to ten. On 10.0%, this expanded further to fifteen. Concurrently, the number overpredicted fell from 44 to 39 and 33, respectively. Severity of this overprediction was notably diminished—with the proportion of substances assigned categories three or four increments higher than their experimental equivalents (for example, Category 5 predicted either as Category 1 or 2) falling at the expense of those merely one or two higher. This may be seen reflected in the general shift from darker to lighter blue shades as evident within Fig. 4. With adoption of the 10.0% threshold, 42 from the 50 compounds were attributed categories either identical to, or one step more conservative than, experimental. By contrast, instances of underprediction, absent entirely at Tier 0, were observed. Quantities, however, were minimal—limited only to a single substance at the 5.0% limit, and to two at 10%.

**Fig. 5:**
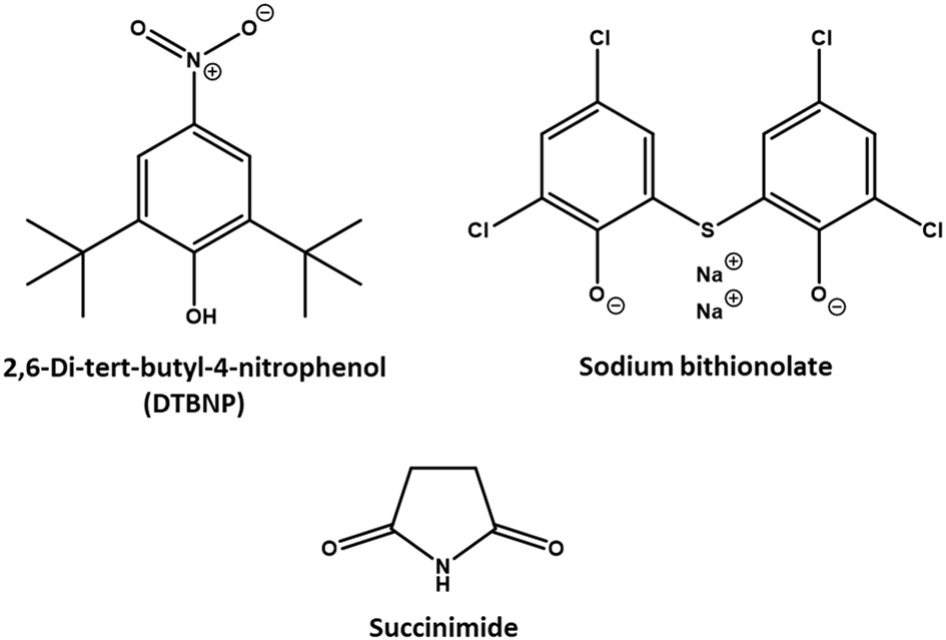
Structures of each of the three illustrative compounds: DTBNP (experimental Category 3), sodium bithionolate (Category 4) and succinimide (Category 5)

To further illustrate the general progression in prediction suitability, specific reference is made to performance against three compounds—each depicted within Fig. 5. These are the Category 3 substance 2,6-di-tert-butyl-4-nitrophenol (DTBNP; ID 4682), the Category 4 sodium bithionolate (ID 2365) and the Category 5 succinimide (ID 6524). Tier 0 assigns each to Category 1—a clear overstatement of measured toxicity. Upon application of the Tier 1 model, figures are revised: 2,6-di-tert-butyl-4-nitrophenol to Categories 2 (5% exclusionary threshold) or 3 (10% exclusionary threshold), sodium bithionolate to Categories 3 or 4 respectively, and succinimide uniformly to Category 4. A visual depiction of the variation in prediction accuracy is presented within Table 9 (being an excerpt from the broader “Predictivity heatmap” present within Supplementary Table 2).

**Table 9:**
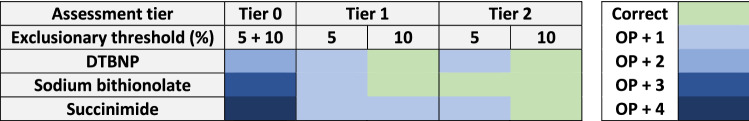
Variation in extent of deviation in category assignment relative to experimentally determined classification for three representative compounds: DTBNP (experimental Category 3), sodium bithionolate (Category 4) and succinimide (Category 5)

Colouration relates to extent of overprediction (OP), defined as differential between predicted and experimental toxicity categories

#### Tier two: in vitro cytotoxicity

Further updating of the Bayesian model through incorporation of in vitro cytotoxicity outcomes served to have only minor impact upon performance, relative to that of Tier 1. At the broadest level, an extremely modest shift away from tendency towards conservatism was witnessed—with the quantity of compounds overpredicted falling from 39 and 33 within Tier 1 (at the 5.0% and 10.0% thresholds respectively) to 37 and 32. These drops were compensated directly by increases in the number of substances assigned to their correct categories, as extent of underprediction remained constant (refer to Table 10).

**Table 10:** Confusion matrices outlining category assignment following application of Tier 2, following application of exclusionary thresholds 5.0% (a) and 10.0% (b)

		Category (predicted)				
		1	2	3	4	5
(a) Category (experimental)	1	5	1	0	0	0
	2	10	4	0	0	0
	3	3	7	0	0	0
	4	0	5	3	2	0
	5	0	3	4	2	1

		Category (predicted)				
		1	2	3	4	5
(b) Category (experimental)	1	4	2	0	0	0
	2	7	7	0	0	0
	3	0	9	1	0	0
	4	0	2	6	2	0
	5	0	0	5	3	2

The described trends were reflected within each of the three exemplar compounds referenced previously, with variation from Tier 1 being either absent in its entirety or instead tending very mildly away from overprediction (Table 9). Category assignments of 2,6-di-tert-butyl-4-nitrophenol remained completely unchanged. Those of sodium bithionolate and succinimide were subject to minor alteration: the former now predicted as Category 4 (with application of either exclusion threshold), and the latter seeing its attribution at the 10.0% threshold switch from Category 4 to 5 (in line with experimentally-derived classification).

## Discussion

Concerns have been expressed over the reluctance of bodies such as regulatory agencies to adopt new approaches in the assessment of chemical safety for human health (Knight et al. 2021; Parish et al. 2020). A key consequence of this has been the continued employment of in vivo protocols, which has in turn, owing to limits imposed by financial cost and availability of expertise, restricted the volume of substances which may feasibly be screened. In many cases, these animal-based laboratory techniques were developed more than 50 years ago. The static nature of this situation indicates in general a lack of confidence in NAM—a position which we postulated may have arisen from inadequacies both in the techniques themselves, and also in the manner through which they have been validated (Mahony et al. 2020).

There was once an expectation that there would be direct one-to-one replacement of existing laboratory animal-based protocols with appropriate in vitro tests (Piersma et al. 2018). However, it has been demonstrated, even in such relatively simple endpoints as localised irritation, that it is beyond the capability of a single assay to cover the range of modes of action which may contribute to ultimate adverse effect (Piersma et al. 2014). The development of Integrated Approaches to Testing and Assessment (IATA) and tiered approaches has seen different sources of information, such as physicochemical properties, QSAR and in vitro methodologies, brought together to ascribe potential for toxicity (Worth and Patlewicz 2016). The outputs from methods combining different data sources exist in the form of probability distributions, not fixed points. Accordingly, they do not lend themselves well to specificity/sensitivity type analysis—in turn making it difficult for regulators to formulate and justify decisions in an objective fashion.

The purpose of this study has been to assess how, through integrating information sourced from various NAM (both in silico and in vitro) into a tiered assessment approach, it might prove possible to enhance overall confidence in the validity of conclusions over those drawn from individual techniques. Acute oral lethality was chosen as the endpoint of interest, owing to its intermediate complexity: whilst many modes of action may contribute, it lacks much of the additional ambiguity inherent within studies of repeat-dose toxicity. Bayesian methodology was adopted as a means through which the information gathered from these approaches might be combined quantitatively, enabling reliable analysis of the contribution added by each to the overall verdict. This was itself expressed in terms of the probability that a substance might belong to one of five categories, each corresponding to defined ranges of LD50.

When considering a range of possibilities, it may be valuable to initially exclude those clearly least likely. Such an approach was adopted in the attribution of toxicity categories to compounds, producing an “exclusionary method”. This proved to have an inherent conservatism, the extent of which could be adjusted through variation of the probability threshold beneath which a category was eliminated. This tendency to overstate hazard is, within limits, an acceptable feature for safety assessments. Within the study, chemicals were assigned to the most severe category holding probability greater than either 5.0 or 10.0%—with the lower value naturally leading to the more conservative judgment. This protocol allowed for the tiered approach to be used with quantifiable certainty, enhancing confidence in decision making and in turn providing objective justification for its adoption. In principle, the methodology could be applied to other areas of toxicity for which a large reference database is present—for instance in determining repeat-dose derived no-effect levels (DNEL) from in vitro studies.

Analysis revealed that the introduction of a second tier of assessment (in silico) imparted substantial improvement in the balance of category assignments, relative to that obtained from the Cramer classification. This is to be anticipated, owing to the fact that Cramer’s scheme was devised for the purposes of a conservative TTC assignment in which the great majority of compounds, with the exception of those most definitively recognised as inert, were assumed to exhibit a uniform toxicity (EFSA Scientific Committee 2019; Cramer et al. 1978). Both the EPA Test method (a series of linked QSARs), and the random forest algorithm (trained using a combination of molecular fingerprint fragments and physicochemical descriptors) contributed greatly to enhancing predictive resolution. These techniques generated estimates at the level of LD50, which could in turn input directly into the Bayesian model. It should be noted that there is potential overlap between the training data adopted in construction of TEST hierarchical clustering and that present within the Gadaleta et al. set. Since scope for improvement existed within the performance of each (*r*^2^ values equal to 0.74 and 0.60, respectively), the further addition of specified molecular structural alerts was considered worthwhile as a route towards addressing the issues arising from presence of outliers. As powerful as the pattern-recognition capacity of QSAR and machine learning might be, this potential can only be realised in the presence of adequate data (Sheridan 2012). As such, it is perhaps inevitable that high toxic potential within compounds possessing unique and complex structural motifs might evade detection. The creation of specific alerts relating families such as trichothecene, saxitoxin, aflatoxin was intended to mitigate this.

By contrast, the in vitro cytotoxicity data contributed little significant information when integrated within the tiered approach—producing only a very moderate redistribution in category assignment. Given the poor correlation between EC50 and LD50, this is very much to be expected. Whilst alternative studies have noted the improved performance of assays such as neutral red uptake (3T3 cells) in capturing in vivo lethality (Prieto Peraita et al. 2013), uncertainties remain concerning the general applicability of the methodology (Schrage et al. 2011). Effective or otherwise, scope for wider use is at present limited on account of the comparatively small quantities of compounds screened. In practice, we were restricted with respect to the data which could realistically be adopted: any candidate system would necessarily be required to cover a significant proportion of the compound inventory set. ToxCast proved the only readily available source matching this description—although the performance of the HEK-293 (human embryonic kidney) viability assay was clearly sub-optimal. This is a predicament which is highly likely to persist until the spread of substances tested through more suitable approaches, such as the aforementioned neutral red uptake screen, is systematically expanded. Shortcomings are even then likely still to remain, owing to the inherent challenges in interpolating organism lethality from the outcomes of simple, two-dimensional cell-based in vitro techniques (Ekwall 1983; Garle et al. 1994). Modes of toxic action such as coagulation impairment and neurotoxicity are, amongst others, liable to be overlooked through such methods—and would perhaps be best accounted for during the in silico assessment phase.

As alluded to with section “Overview of tiered approach”, a further tier (Tier 3), may be introduced to incorporate the targeted application of in vivo testing. This would be considered necessary only if there was large uncertainty with respect to the final verdict, or there existed an imperative to otherwise challenge the assigned category. This might arise for commercial reasons—for example, if an important product was to be attributed a category suggesting severe toxicity, in turn necessitating its exclusion. Alternatively, there may be concerns that a chemical had been predicted as low toxicity, despite the presence of additional evidence suggesting that it might in fact present a greater hazard. In these cases, it would not be necessary to carry out a full assessment of LD50. Instead, a refined means of assessing acute lethality, such as the OECD Guideline 425 (OECD 2008), could be employed. In brief, this is a stepwise procedure utilising single animals, whereby the first receives a dose marginally below the best estimate of the LD50 (which would itself be derived from Tier 2). Depending on the outcome for the previous animal, the dose for the next is increased or decreased—usually by a factor of 3:2. This sequence continues until there is a reversal of the initial outcome (i.e., the point where an increasing dose results in death rather than survival, or decreasing dose results in survival rather than death); at this stage, additional animals are dosed. The exclusionary method adopted within this study could be used to limit the use of further animals if no response is seen at a dose which would provide a level of confidence indicating that a particularly severe category of concern could safely be excluded.

Our investigation has indicated that Bayesian methodology can be used in the development and evaluation of tiered approaches for acute lethality assessment. Accordingly, the question arises of how successful such a technique might be if applied to alternative manifestations of toxicity. Previously, Bayesian analysis has been employed in the integration of data describing drug-induced liver injury (Semenova et al. 2020; Williams et al. 2020), cardiotoxicity (Felli and Leishman 2020) and skin sensitisation (Reynolds et al. 2019). Whilst potencies in many toxicological endpoints may be expressed readily within categorical terms (this format being particularly amenable to Bayesian treatment), there remain those for which such framing is not so practical (National Research Council 2014). Although it is comparatively trivial to assign bands of potency to points of departure, it is more difficult to imagine how ranges of effects could similarly be categorised. The idea of “protection not prediction” challenges whether the “free form” recording we currently have of adverse effects is necessary. Would it be sufficient simply to categorise outcomes as “severe”, “moderate” or “mild” in order for risk assessment and risk management to operate? In more general terms, use of the tiered approach is dependent upon both the sourcing of large inventories containing outcomes of conventional laboratory animal-based protocols, and on the acquisition of NAM data (in the form of relevant in vitro endpoints, QSAR etc.) which may readily be integrated across the various stages of assessment. Some difficulties were experienced in accessing data on account of the varying formats within which it may be held. Agreement on the formatting of databases containing in vivo and in vitro outcomes would enable the development of methodology in which regulatory authorities could have confidence.

In conclusion, we have shown that Bayesian inference can be adopted to integrate data from a variety of NAM sources—producing output from in the form of probability distributions which may subsequently be used in provision of objective assessments of toxic potential (in this instance, acute oral lethality). An exclusionary approach enabled assignment of chemicals to established toxicity categories, with a defined level of certainty. In terms of validation, this has potential to offer increased confidence relative to the simple quantification of sensitivity and specificity. Whilst the remit of this particular investigation has remained limited to the relatively narrow area of acute lethality, there is great scope for wider application of the approach. Accordingly, it is our intention that the demonstrated methodology might ultimately serve in providing a level of confidence in NAM sufficient to gain wider use in regulatory decision making—offering as it does the benefit of reducing the number of laboratory animals used, in turn allowing more efficient assessment of chemicals within existing financial and expert resources.

## Supplementary Information

Below is the link to the electronic supplementary material.

Supplementary file1 (XLSX 1680 kb)

Supplementary file2 (DOCX 43 kb)

## Data Availability

The authors confirm that the data supporting the findings of this study are available within the article and its supplementary materials.
