# Supplementary material for: The use of Bayesian methodology in the development and validation of a tiered assessment approach towards prediction of rat acute oral toxicity
Source: Arch Toxicol. 2022 Jan 16;96(3):817–30. doi: 10.1007/s00204-021-03205-x (PMC8850222; doi:10.1007/s00204-021-03205-x)
Supplement: Supplementary file 2 — Supplementary file2 (DOCX 43 kb) [file 204_2021_3205_MOESM2_ESM.docx]

**The Use of Bayesian Methodology in the Development and Validation of a Tiered Assessment Approach towards Prediction of Rat Acute Oral Toxicity**

**Archives of Toxicology**

James W. Firman,^1*^ Mark T.D. Cronin,^1^ Philip H. Rowe,^1^ Elizaveta P. Semenova^2^ and John E. Doe^1^

^1^School of Pharmacy and Biomolecular Sciences, Liverpool John Moores University, Liverpool, United Kingdom

^2^Department of Mathematics, Imperial College London, London, UK

^*^Corresponding author ([j.w.firman@ljmu.ac.uk](mailto:j.w.firman@ljmu.ac.uk))

**Supplementary material**

**Supplementary Table 4:** Overview of structural alerts present within ten or fewer compounds. Depicted is the defining structural fragment, alongside details relating to the known toxic mechanism associated with the class.

| **Alert title** | **Defining structure** | **Description** |
| --- | --- | --- |
| **Aflatoxin** | **** | Hepatic activation forming reactive metabolites |
| **Bromethalin-like** | **** | Neurotoxicant  (inhibition of oxidative phosphorylation) |
| **Ochratoxin** | **** | Induction of nephrotoxicity |
| **Saxitoxin-like** | 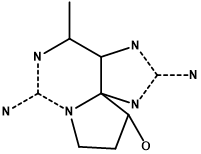 | Neurotoxin  (Na^+^ channel blockade) |
| **Strychnine-like** | **** | Neurotoxin  (glycine receptor antagonism) |
| **Trichothecene** | **** | Inhibition of protein synthesis  (ribosomal) |
| **Vitamin D** | **** | Induction of hypercalcaemia |
| **Vitamin K antagonist (indandione)** | **** | Anti-coagulant |
